# Deep Learning Model for the Detection of Real Time Breast Cancer Images Using Improved Dilation-Based Method

**DOI:** 10.3390/diagnostics12102505

**Published:** 2022-10-16

**Authors:** Theyazn H. H. Aldhyani, Rajit Nair, Elham Alzain, Hasan Alkahtani, Deepika Koundal

**Affiliations:** 1 Applied College in Abqaiq, King Faisal University, P.O. Box 400, Al-Ahsa 31982, Saudi Arabia; 2 School of Computing Science & Engineering, VIT Bhopal University, Bhopal 466114, India; 3 Computer Science Department, King Faisal University, P.O. Box 400, Al-Ahsa 31982, Saudi Arabia; 4 School of Computer Science, University of Petroleum & Energy Studies, Dehradun 248007, India

**Keywords:** AlexNet, accurate detection, convolution neural network, deep learning, dilation convolution, data augmentation, VGG16 network

## Abstract

Breast cancer can develop when breast cells replicate abnormally. It is now a worldwide issue that concerns people’s safety all around the world. Every day, women die from breast cancer, which is especially common in the United States. Mammography, CT, MRI, ultrasound, and biopsies may all be used to detect breast cancer. Histopathology (biopsy) is often carried out to examine the image and discover breast cancer. Breast cancer detection at an early stage saves lives. Deep and machine learning models aid in the detection of breast cancer. The aim of the research work is to encourage medical research and the development of technology by employing deep learning models to recognize cancer cells that are small in size. For histological annotation and diagnosis, the proposed technique makes use of the BreCaHAD dataset. Color divergence is caused by differences in slide scanners, staining procedures, and biopsy materials. To avoid overfitting, we used data augmentation with 19 factors, such as scale, rotation, and gamma. The proposed hybrid dilation deep learning model is of two sorts. It illustrates edges, curves, and colors, and it improves the key traits. It utilizes dilation convolution and max pooling for multi-scale information. The proposed dilated unit processes the image and sends the processed features to the Alexnet, and it can recognize minute objects and thin borders by using the dilated residual expanding kernel model. An AUC of 96.15 shows that the new strategy is better than the old one.

## 1. Introduction

Cancer is now the main cause of mortality in almost every country. Cellulitis is a condition characterized by abnormal cell development. Tissues are made up of cells that collaborate with one another. Diseased cells are replaced by healthy cells. Tumors and cancers are uncontrollable cell growths that have shifted from their native environment. These are the most prevalent cancer kinds [1]. Pathologists employ histology to assess the progression of cancer in organs [2] By putting together a histological tissue slide for microscopy. Histopathological specimens include a variety of tissues that surround cells and structures. It takes time to physically understand historical images. Many years of expertise in computer-aided image analysis have the potential to increase histopathological pictures’ analytical and predictive capacities. A trustworthy second opinion enables histopathologists to do more.

This may aid in the diagnosis of the problem. In 2012, cancer claimed the lives of 8.2 million individuals, and 8.8 million in 2015. Breast cancer claimed the lives of 5.7 lakh individuals in 2015. Between 2005 and 2015, the global cancer incidence increased by 33% [3]. We were able to give each of the images a uniform appearance by utilizing a basic H&E color normalizing procedure that we obtained online. The photographs were then scaled up to four distinct sizes, each with a different aspect ratio of one, a half, a third, and a quarter. These enlarged images were separated into four identical, non-overlapping halves. Individual patches were made without any augmentation, then stitched together by increasing each patch to a total of 224 by 224 pixels. We were able to obtain exactly what we needed. Our team created an enhanced patch-wise dataset on top of a concatenated subset of four non-overlapping neighboring patches. Pathologists are trained to analyze histological images from several perspectives, since staining and recording can take place in a variety of conditions. To mimic the actions of a pathologist during an examination and represent real-world variation, we used a variety of data augmentation techniques, such as horizontal and vertical flipping, rotation, shifting (in both dimensions), brightness, magnification, and blurring. The idea was to create a scenario equivalent to that of a pathologist employing these techniques to carry out their work. The dataset’s integrity will not be jeopardized in any way, even if its size increases as more data are uploaded to it. In a published study, the use of data augmentation and patching methods for histological classification was shown to be theoretically feasible. This argument is reinforced by recently released study data. Once an agreement was achieved, each of the newly created patches was given a name that matched the category of the original picture that had been utilized in the creation process. After training using image-net weights, the model compared the values it had given to patches of varied sizes. This was completed after the model had been trained.

A patch-wise classifier evaluates multiple unique parts of the image at various magnification levels, before selecting how to categorize the full image. The results of this processing will be combined with the results of processing the whole picture patches in the following step to produce an image-wise classification. This classification will be determined by the total number of image patches under development. To accurately categorize histology photographs into one of the various required classifications, first, one must find the key components of the images and then study these features. Only then will it be possible to properly label the pictures. The data supplied describe the nucleus characteristics, texture, and overall tissue architecture. The shape, color, and structure of the nucleus can be used to identify and distinguish cancer cells from healthy ones. Normal cells can be identified by the absence of specific characteristics. This stage is critical for distinguishing between benign and malignant cells (e.g., density or variability). A fundamental understanding of tissue architecture is required to distinguish between in situ and metastatic carcinomas. The categorization method is used to generalize learned traits over a wide range of spatial sizes, from glands to cell nuclei.

Cancer is the leading cause of mortality among Indians. In 2016, only one new instance of breast cancer was discovered and identified in India. Since more people have been diagnosed with cancer in the last 10 years, experts expect that the overall number of cancer patients in India will rise by 2025. Breast cancer is the leading cause of mortality for women under the age of 50, and it ranks second on the list of the world’s deadliest diseases. Early detection has been shown to have a high association with public awareness [4], and both variables can dramatically reduce death rates. If a patient’s prognosis is positive and they obtain an accurate diagnosis, they have a far greater chance of fully recovering. It is, therefore, critical to develop effective technologies that can increase the rate of early diagnosis and reduce death rates. However, histological examinations continue to be the most used imaging tool for detecting breast cancer [5]. Pathology diagnosis in its current condition is highly interpretative. Even in ideal settings, this operation is time-consuming and demanding; one can imagine how much more difficult it is when the pathologist is weary or under pressure at work. Tissue samples obtained during biopsies or surgical procedures are evaluated to determine whether the patient has cancer. These samples are inspected under a microscope. The operation is carried out by pathologists, who are highly experienced medical professionals. Histology, on the other hand, is a method that needs to be performed quickly, even though it is a difficult process and takes a long time. Following treatment, the tissue is sliced into small sections that are thin enough to enable light to pass through them, allowing light microscopy to be performed on the sections after they have been cut. Although frozen section histology can be utilized for intraoperative assessment, it is a labor-intensive and time-consuming technique. The tissue can only be studied using exceedingly small samples. The use of computerized image inspection and machine learning algorithms has recently become practical. A decade ago, digital pathology used microscopes with cameras to digitally scan complete tissue samples. Recent advances in image analysis and processing power have assisted in the incorporation of computer-aided diagnostic (CAD) systems into pathology lab operations. These tools were made to help pathologists find, diagnose, and predict illnesses. In terms of diagnosis and prognosis, DL-based CAD systems have made major contributions to the growth of the medical business. Data from radiology, pathology, cardiology, pharmacology, cancer, and genomes are analyzed by these systems. In recent decades, more sophisticated systems based on machine learning principles have been used to diagnose cancer. Immunohistochemistry is one such approach. There has been a substantial increase in the number of times this strategy has been deployed. Since it is beneficial in cancer prediction and prognosis, DL is generally recognized as one of these approaches. The higher diagnostic accuracy of DL can aid in the improvement of mammograms, ultrasounds, and digital breast tomosynthesis (DBT). Because it provides a better degree of precision than other approaches, DL is used in the treatment of breast cancer (BrC). There has been a flood of scientific articles pertaining to BrC during the last several years. Deep learning is used in several contexts in all these studies. Deep learning algorithms are able to automate the BrC diagnosis procedure, which is riddled with flaws and errors. Despite the large number of BrC classification review studies that have been conducted thus far, only a small number of them can offer guidance to future researchers. Even though these articles give a rather complete literature overview on BrC, they may benefit from including a few additional parts on deep learning. An example sentence is as follows: the great bulk of BrC literature evaluations have focused on either classic ML techniques with broad strokes. Both approaches involve feature extraction at various stages of the diagnostic process, which is comparable. Deep-learning architectures, such as generative adversarial networks, extreme learning machines, and similar systems, were unable to generate a BrC diagnosis. Recent BrC research has led to the development of several imaging modalities. However, most prior review studies ignored emerging imaging modalities, such as infrared thermal imaging, digital breast tomosynthesis, and computed tomography. There are review articles on digital breast tomosynthesis [6], but they do not include all imaging modalities that can be used to classify BrC. This is due to the article’s structure, which limits what can and cannot be included. They also failed to provide a clear explanation of the advantages and disadvantages of previous research, which impeded an otherwise fascinating analysis of deep learning-based approaches. They also did not give a full explanation of the positive or negative aspects of the previous investigations.

Automatic biological data analysis may help pathologists make early or on-the-spot diagnoses. These strategies assist pathologists in reducing their workload by isolating and screening out non-cancerous regions. They also assist pathologists in detecting breast cancer early, which reduces fatalities. An assessment of the research paradigm, strategy, or framework is required. In a validated system, performance measurements for segmentation and classifiers may be employed. As a result, two datasets must be used for training and testing. The system must be evaluated on a separate dataset to prevent memorization.

## 2. Related Work

In this part, we will discuss important work in medical image assessment for breast cancer diagnosis, as well as machine learning and deep learning models. It has been important to improve the architecture of the best deep learning models for detecting cancer in the last few years.

### 2.1. CNN’s Infrastructure

Convolution neural networks (CNN) impose strain on neural networks, which have been demonstrated to be useful in breast cancer categorization and diagnosis. ConvNets, named accordingly because of the veiled layers they include, suggest inscriptions. Convolution, pooling, and a fully linked layer comprise a CNN [6]. Convolution collects local picture properties, pooling decreases dimensionality, and a fully connected layer generates the desired output. Brighter pixels can be used to show picture borders and emphasize local image attributes for more processing. Convolution is useful because it involves both these tasks.

Each layer of the network’s foundation contains an activation function, as well as convolution and pooling functions. Convolution takes one image and one filter as input and outputs the image. The image size (128 × 128 × 3), image height, width, and channels, for example, all influence the neural network’s performance. The image channel has an RGB image channel and a height of 128 × 128 pixels; the processing size is 49,152 bytes (128 × 128 × 3). For example, if the image is 2048 × 2048 × 3, a large, weighted extent of 12 million is required. A typical neural network, such as a CNN, may overfit very small images, waste memory, and require extensive image processing.

(1) $$C=\sum_{1}^{i}\sum_{1}^{j}I_{ij}F_{ij}$$

**Figure 1:**
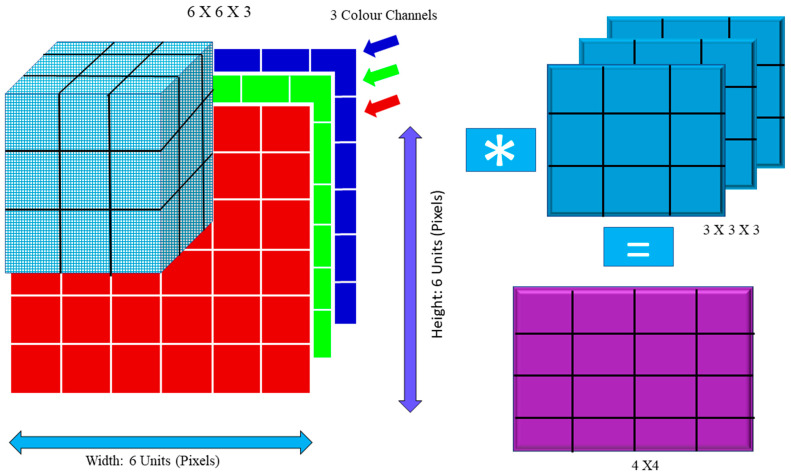
Convolution Operation: Image Matrix Multiplies Kernel or Filter Matrix.

*F* denotes a convolution kernel or filter, whereas *I* *j* denotes rows and columns. For example, in Figure 1, multiplying the image by the kernel the figure shows both the input image and the filter. The new two-dimensional output is shown in the figure. Convolution breaks the image down into perceptrons, which are subsequently flattened on a (y) X axis (z). Each layer has *x* filters for locating characteristics. Layer *L* generates X-sized feature maps that are annotated as follows:

(2) $$C_{i}^{L}\;=B_{i}^{L}+\sum_{j=1}^{x^{\left(L-1\right)}}F_{i,j}^{L}*C_{j}^{\left(L-1\right)}$$

where $B_{i}^{L}$ is the bias matrix and $F_{i,j}^{L}$ is the filter that connects the *j*th feature map in the layer.

For instance, the blue channel may be −1, but the other channels could be +1 or 0. To compute the convolution value, one must use the dot product. The images are distorted by convolution. In addition, (Is) = 1 in this example. If (Is) and (Is), the picture size is the same (Fs).

Cs = ((Is − Fs)/S) + 1 (3)

If the values of Is, Fs, and S are 6, 3, and 1, respectively, then Cs = (6 – 3 + 1) = 4. The following equation demonstrates how the output dimension was calculated:

(4) ⌊(Width−Fs+2P)/S]+1⌋

The filter size is represented by Fs, padding is represented by P, stride is represented by S, and Equation (4) is the floor value.

**Figure 2:**
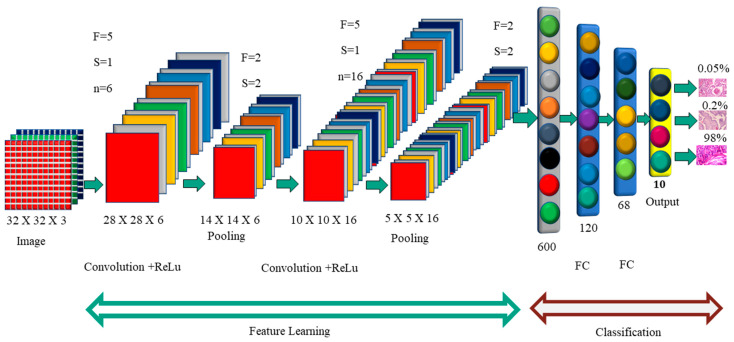
Convolution Neural Network Architecture.

Figure 2 shows how each image was convoluted before pooling. The activation function is used by the F.C. layer to categorize the cancer image. Convolutional neural networks have a variety of named architectures that are used in the detection and classification of breast cancer. A convolutional neural network structure, in its most basic form, is a series of layers that transform input data into a classification score. The efficient layers used in the CNN building include the convolution layer, pooling, ReLu, and fully connected layers. In the next section, we will discuss the CNN structures that are used in breast cancer in greater depth.

#### 2.1.1. AlexNet Architecture

**Figure 3:**
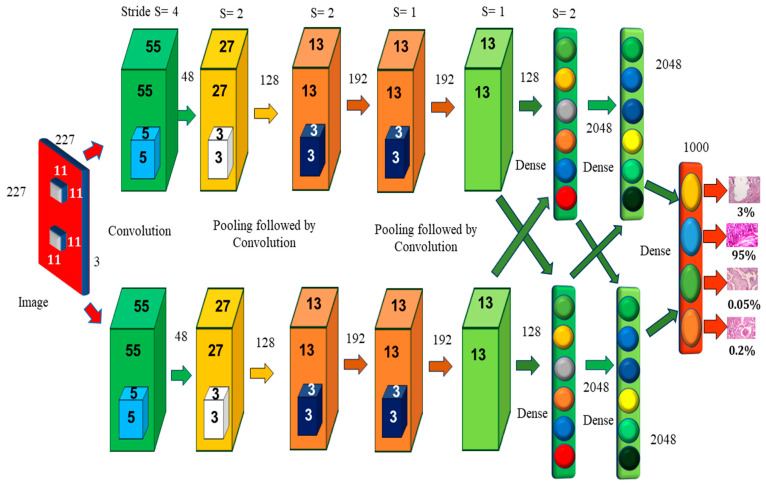
AlexNet Architecture [10].

Alexnet’s learning parameters include 60,000 neurons and 6 convolutional layers (Figure 3). Two GPUs may be used to run the network, which speeds up training. The original image has the dimensions of 224 × 224 × 3, but padding lowers it to 227 × 227 × 3. Each convolution layer consists of a convolution filter followed by the ReLu activation function, which enables AlexNet to accelerate deep network training to be five to six times faster than other activation functions, such as sigmoid or tanh [7]. The max-pooling process is used to reduce the dimensionality and downsample the image or data. Because of the overlapping nature of Max Pool, AlexNet uses a 3 × 3 pooling window and a stride of 2, which reduces inaccuracy when compared to the non-overlapping window of size 2 × 2 and stride 2 and avoids the blurring difficulties that average pooling often produces [8]. CNN assesses various variants of the same image and assists in the prevention of overfitting. Instead of a 256 × 256 image, AlexNet uses a cropped 227 × 227 image. When a neuron dies, the whole connected network may help the network retain the learned weight [9]. It prevents overfitting, since neurons that have been removed do not participate in forwarding or backward propagation. In local response normalization, group convolution is used to reduce time and create a layer that is completely connected with the same number of groups for both input and output channels.

#### 2.1.2. VGG16 Architecture

**Figure 4:**
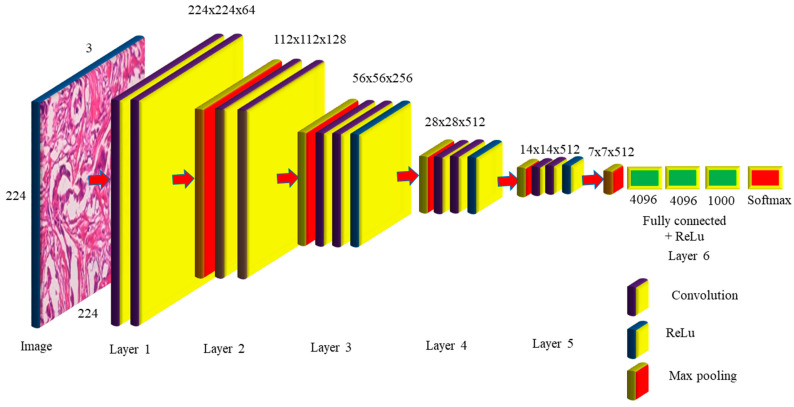
VGG-16 Network Architecture Map.

When the size of the network is large, the deep neural network performs well. The VGG networks built by the Visual Geometry Group include A, A-LRN, B, C, D, and E. VGG−16 networks C and D have 13 convolution layers, 3 completely connected layers, and 16 layers, respectively, as shown in Figure 4. The C network employs a 1 × 1 filter size for convolution, but the D network does not. Training is necessary for the 134 million parameters, and since network D usually employs a 3 × 3-sized filter for convolution operations, 138 million parameters must be taught. Because it includes 16 convolution layers followed by 3 completely linked layers in 19 levels, network E is known as the VGG-19 network. All VGG networks employ ReLu but do not use local response normalization, since it takes up more memory space and requires more training time [10]. The primary distinction between AlexNet and VGG is that AlexNet employs an 11 × 11 kernel with a stride of 4 × 4, while VGG employs a 3 × 3 kernel with a stride of 1 × 1. VGG generates a 1 × 1 convolution filter that aids in prediction and categorization. The network improves the data by using a multiscaling technique that increases the number of inputs and eliminates the overfitting problem [11]. The network’s main difficulty is that training takes a long time, and the network’s weights need more space and bandwidth [12].

The VGG-16 network takes 224 × 224 pictures as input, with 3 channels guaranteed by 2 convolutions of 64 kernels in layer one, and stride 2 × 2 was used to lower the dimension by 50% in the max pooling with 128 channels, followed by 2 convolution layers in layer two, as observed in Figure 4. Layer 3 consists of a pooling layer, followed by 3 256-channel convolution layers; layers 4 and 5 execute max polling and 3 512-channel convolution operations. Layer 6 is completely linked and is made up of 2 1 × 1 × 4096 neurons, followed by a group of 1000 neurons. These are sent to the Softmax layer, which uses them to create classification values. The VGG-16 network hidden layers, such as AlexNet, employ ReLu to make the VGG-16 more efficient and quicker. The number of trainable parameters required in layers 1 to 5 is 36,844, 147,456, 589,824, 2,359,296, 2,359,296, respectively. The completely linked layers need 102,760,448, 16,777,216, and 4,096,000 trained parameters for a total of 138 million. The VGG16 model, which had previously been pre-trained with CCN deep learning, was used to extract the breast image attributes. To do this, we must change the VGG16 graph by eliminating the most recent collection of completely linked layers. These data were then disseminated to several classifiers for processing. A thorough examination of the publicly accessible tests on the Breakhis dataset was carried out, and a set of performance criteria was developed to assess the findings (of the test data portion). The findings of the research outperformed those of several other tactics that are now leading the way in innovation.

Each year, breast cancer claims the lives of hundreds of people [13]. Cancer prognosis and deep learning remain major concerns in the health sciences. Machine learning contributes to the advancement of technology. Early detection improves the chances of survival. Deep learning can predict cancer [14]. A variety of factors promote cancer development. Research that included women who had their first child later in life was carried out. A bulge and fast cell separation were reported [15]. There are three forms of fibroadenomas, which are as follows: phyllodes, fibroadenomas, and mucinous carcinomas. The BreakHis collection contains images of benign adenosis, ductal carcinoma, lobular carcinoma, mucinous carcinoma, and papillary cancer. Deep learning aided in image categorization improvement [16]. The CNN and BiCNN models correctly identified 90% of breast cancer patients. Deep learning can detect mitosis and metastases [17,18,19] in histological pictures to diagnose malignancy [20]. A variation in the appearance of an identical H&E image of a cancer tissue sample was identified. Cell desegregation in imaging is difficult without feature evaluation. It borrows features from the VGG16, and a neural network discovered that AlexNet’s large color images perform well. Inception v3, a deep learning model, detected lung cancer [21]. In H&E pictures, the nuclei are shown in blue/purple, whereas the cytoplasm is shown in pink. The maximum core images may show variations in noise, texture, intensity, and form. AlexNet addresses gradient dispersion via ReLU non-linearity. Deep CNN training may also be accelerated using Tanh or Sigmoid saturation functions. Dropout is used to modify the architecture of the cell, while max pooling is utilized to minimize the blur. There are now 63 million AlexNet parameters in total. After padding, the picture measures 227 × 227 × 3. The activation of Softmax gives 1000 categorization values. VGG16, as is the case with AlexNet, has 16 layers and an error rate of 7.7 percent. There is no such thing as normalization of local responses. VGG computes stride 1 using 3 × 3 kernels, while AlexNet uses 11 × 11 kernels. Shen and his colleagues used a max-pooling layer that followed five convolution units. Smaller pictures with more channels were obtained and each tier generated 100 neurons.

## 3. Proposed Model

It gathers multiscale contextual data, while maintaining image quality and without increasing the number of model parameters. Spatial resolution is reduced when convolutions are dilated. Gridding of dilated convolutional networks has already begun. In this section, the preprocessing and the working principal of the proposed model are given.

### 3.1. Data Augmentation

**Figure 5:**
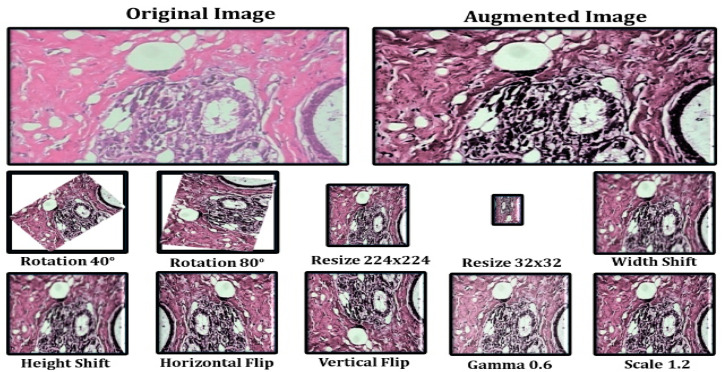
Data Augmentation Results.

The model’s initial goal is to deal with overfitting concerns caused by the restricted number of photos used in the training phase. To address this, we employ data augmentation as the first step, so that we may make use of rotation, scaling, shift, and gamma correction with various parameters. Breast Cancer Histopathological Image Classification (BreakHis) includes 2480 benign photos and 5429 malignant images, whereas Breast Cancer Histopathological Annotation and Diagnosis (BreCaHAD) includes 162 breast cancer images. Techniques such as flipping, rotating, shifting, scaling, and gamma are all valuable. The parameters tested for augmentation are shown in Table 1. In the top row of Figure 5, a histology picture from the dataset is shown, followed by a strain normalized image and a rotational image. The second row shows a flipped image with gamma values between 0.3 and 1.2.

Data augmentation increases the number of images in a dataset, which eliminates overfitting issues caused by dataset size restrictions [21,22,23]. Figure 5 depicts the picture enhancement outcome. The histology picture in the dataset is followed by a strain normalized and rotational image. The second row has an inverted picture with gamma values of 0.2, 0.5, 0.8, 1.0, and 1.2. Image augmentation is the process of rotating, scaling, and flipping images. The photos have been scaled by 0.4, 0.7, and 1.3. Using the reflections, one can rotate the picture. 

### 3.2. Hybrid Dilation Deep Learning Model

**Figure 6:**
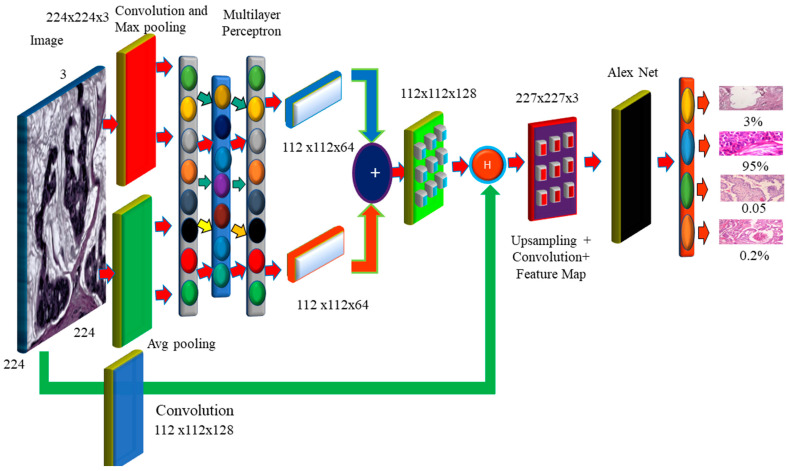
The Proposed Hybrid Dilation Deep Learning Model.

Histology images were utilized to evaluate the correctness of the pre-models. Using this technology, cancer cells are captured and differentiated. The proposed model decreases network overfitting, while improving accuracy, as shown in Figure 6. The missing pixels are then dilated and the Hadamard product is used to connect the input result to the entirely connected layers. The technology improves the accuracy, while broadening the receptive field. To extract features, dilated convolution is employed. Dropout and overfitting are reduced when data are normalized in bulk. Excellent results in terms of localization and mitotic centroid measurement are achieved. All cancer cells need high-resolution images. The suggested architecture makes use of fewer than half of the network layers shown in the picture. Batch normalization of a simplified symmetric skip network is carried out. The proposed model has the highest AUC (96.15). In addition, 3 × 3 convolution aided in the identification of micro-cells.

In this section, we present some of the most pressing issues and solutions. In the absence of visuals, convolutional neural networks overfit, impairing detection. The model provides two preprocessing processes for extending the dataset. First and foremost, it combines BreakHis and BreCaHAD. The dataset is then rotated, shifted, gamma-flipped, and scaled. While the degree of malignancy remains the same, the manifestation of the disease is altered. This method improves photographs by adjusting the contrast without affecting the image data.

We can look for minute variations in histology images to predict breast cancer. It is recommended that a deep learning breast cancer screening system based on photographs is used. Our key scientific contribution is the recognition of breast tumors in histopathology photos using deep learning. Breast cancer categorization considers overfitting and color imbalance. Data expansion is currently ongoing. This model can detect low-level edge, contour, and color characteristics. There is less duplication, and ghost models are more straightforward. It depicts the model’s performance without sacrificing visual information. Breast cancer may be discovered by mammography, PET, biopsies, ultrasound, and magnetic resonance imaging. Therefore, this therapy might be advantageous. A pathologist must study the photos to establish if they are benign or malignant. CNN enhances deep learning systems for identifying breast cancer by using a cancer cell of grade 3 and its prognosis. The CNN model’s multilayer architecture identifies the cancer [24,25,26]. Convolution captures the local characteristics, pooling reduces dimensionality, and a fully connected layer produces the desired outcome. Convolution can present the edges and local visual elements. Normalization, pooling, and convolutional layers are all types of terminology used to describe the normalization, pooling, and convolution processes. The picture of the CNN layer is 128 pixels wide and has 3 channels (128 × 128 × 3). Small image databases need less memory and compute power. Pixels have an influence on algorithms in the extraction of characteristics from histology pictures. As a result, we employ normalized and improved photos to find microscopic cells. The smallest cancer cells and thin cancer cell borders may be found via max pooling. It selects a value at random from each receptive field. Assume that one wants to apply a filter that creates a pooling layer on a 3 × 3 pixel square image. One-pixel rotations in both the horizontal and vertical directions have little influence on the outcome of the pooling layer. One must remember that this applies whether you apply it to the original layer or to a modified version of the image, such as the one created after applying a convolution layer to the original. The pooling layer comes after the convolution layer and is often the next stage in the process. Despite the convolution layer’s effectiveness in detecting a meaningful structure within a sample of the data, the pooling layer introduces more noise into the equation, making it more difficult to localize the structure to a precise place. This is because the convolution layer handles the first stage of structure recognition. By offering an abstracted form of the representation, max pooling is one strategy for preventing model over-fitting. The main motivation is to increase the model’s accuracy. This decreases the number of facts that must be memorized and, as a result, the amount of effort necessary. Furthermore, the internal representation is translationally invariant. Because the total number of parameters has been cut down, translation invariance can now be maintained. The preprocessing phases of strain normalization and data augmentation increase feature extraction [27,28,29]. Many histology images with the same label may have different pixel intensity levels. This has an adverse effect on performance. This problem has been solved by the proposed feature extraction module, which takes the input image and performs the convolution procedure. Those used in histopathology frequently have a slightly greater resolution and cover a significantly larger area than images used in other imaging disciplines. A typical pathology slide’s resolution can exceed 100,000 pixels wide and 100,000 pixels deep, when scanned at high magnification. This is possibly the most distinguishing feature of histology photos. Because clinical annotations, such as written reports, apply to the entire picture or collections of images rather than specific locations within the image, it may be difficult to precisely match a search “query” with the relevant section of the image. Therefore, it may also be difficult to locate the region of the image that matches the “query” that was entered in the search field. This is demonstrated by the fact that a tumor in a pathology imaging study may be just one hundred pixels wide, or around one millionth of the whole picture area. Even if a medical specialist, researcher, or medical student found the photos using a text-based search, physical inspection would still be required to diagnose the lesion. One must complete this stage before proceeding to any other form of analysis. Real-world pathology cases, such as those in many other professions, typically involve multiple photographs. The text labels that are given may not be specific enough for the illness subtype that is of interest.

Reverse image search, also known as content-based image retrieval, looks for images that are visually “similar”. This strategy might be applied to address concerns outside of medicine. A clinician may opt to search a database for identical lesions as part of the diagnostic procedure to confirm whether a given trait of interest is suggestive of a benign or malignant histologic mimic, such as basal cell carcinoma. This approach can be used to determine if a worrying discovery is a benign or malignant histologic mimic. The doctor may assess the affected location to determine whether the characteristic of concern is a benign or malignant histologic mimic. Examples of technology that may have applications outside of the medical area include “search by photo” for common images, visual search for retail products, and various tools for faces and art. Analogous research exists in the medical imaging-related subfields of pathology and radiology. Models were created for each application in early CBIR systems that relied on machine learning. So, in order to install these technologies, it was necessary to collect labeled data for each application, which took a significant amount of time. Dimensionality reduction has two components, Fx and Fy. Fx then goes through average pooling to obtain smaller Fx (avg) and max pooling Fy (max) values to obtain a better value, which is then combined to make the feature map.

Feature maps are dilated with a dilation value of 4 to increase the receptive field of the architecture. As the dilation value increases, the receptive field grows, and the dilation mechanism multi-scales the features. The features are then obtained by performing element-wise multiplication of the input image and the feature map. The feature is then aggregated with the features generated by global average pooling and maximum pooling, followed by fully connected layers and dilation, and the weighted sum of these two blocks is used to retain the features of both long and short regions, enhancing the essential elements, while suppressing the useless information. As a result, the pixels can be observed more clearly. Furthermore, the cancer cell may be detected more precisely.

Expansion convolution searches for items while minimizing complexity (Perone, Calabrese & Cohen-Adad, 2018). Dilation broadens the network rather than increases the size of the filters at all levels. The filter depth may be calculated using the formula (2d + 1) × (2d + 1). The four receptive fields for d = 1, 2, 3 are 3 × 3, 5 × 5, 7 × 7, and 9 × 9. In addition, (x, y) = I (x + s × I y + s × j) denotes the dilated convolution (7). D(x, y) is a sparse dilated convolution filter with the input parameter I(x, y). When s = 1, dilation and standard convolution provide identical results. Dilation widens the receptive field without increasing the number of parameters. It also makes the process easier because dilation only adds a convolution operation to the process.

## 4. Experimental Results

This section evaluates the deep learning model. We begin by comparing the model’s performance to other models.

### 4.1. Trial and Evaluation of Models

**Figure 7:**
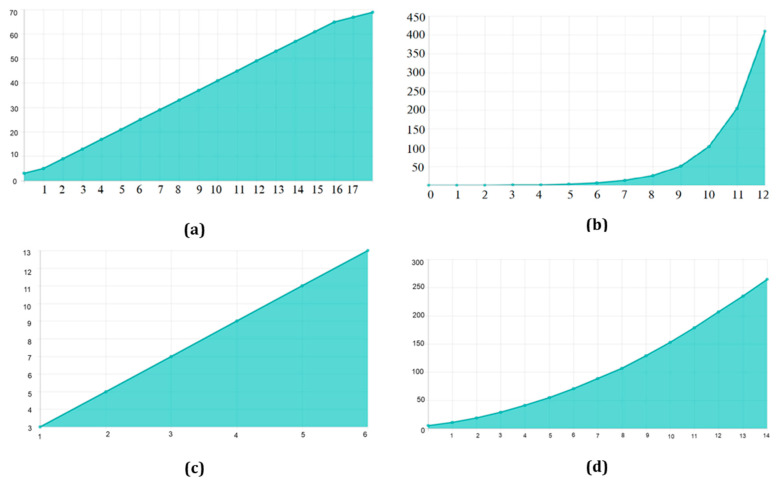
Receptive Field Growth Layer vs. Receptive Field Number: (**a**) Stride (S) = 1, dilation 1, 1, 2, 2, 2 (**b**) S = 2, dilation 1, 1, 2, 2, 2 (**c**) S = 3, dilation 1, 1, 2, 2, 2 (**d**) S = 4, dilation 1, 1, 2, 2, 2.

There are 70 and 30 training and testing datasets, respectively. In addition, we update both the training and enriched data in 80/20 splits. The experimental data sets are summarized in. Table 2 contains image information for both datasets. Table 3. They are used to configure the network and choose hyperparameters. The proposed model is used for object recognition. Convolution was applied after max pooling to increase the receptive field, as shown in Figure 7.

### 4.2. Testing

TensorFlow 2 and Keras 2.4 are used in this Python 3.9 model. It has two Nvidia GeForce GTX 2070 GPUs, as well as a 16 MB cache. A dilated convolution model with many scales is used for object identification. Convolution was applied after max pooling to increase the receptive field (Figure 7). Dilated convolutions are the most dominant. The proposed multi-scale extended convolution model is better at predicting pixels for both malignant and benign tumors than the previous model.

BreakHis comprises 7909 actual samples (photos) from 82 people, including 2480 benign and 5429 malignant photographs, as well as 162 BreCaHAD breast cancer images. There were 153,349 parameters in the 7909 BreakHis samples and 3078 BreCaHAD images. The sections that follow compare the results of binary and multiclass classification. This model excels at identifying small details. Convolution was used to enhance the receptive field after max pooling. Instead, dilated convolution is used in the suggested model. The suggested model differentiates normal and malignant cells. Other convolution layers, as well as stochastic units for downsampling and upsampling, are used in the new model.

Better specificity, accuracy, precision, recall, and F1-score are shown in Table 3. The proposed model has a 98.60 percent accuracy for BreCaHAD and a 98.41 percent accuracy for BrekHis in the model with enhanced images, where A is the original picture and B is the enhanced image.

**Figure 8:**
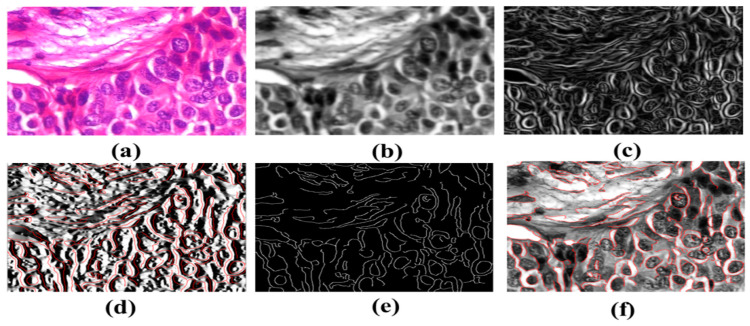
Detection of Breast Cancers. (**a**) Input H&E Image (**b**) image after gaussian smoothing (**c**) It is the gradient magnitude it is determining in which direction the change in intensity is pointing (**d**) Gradient in the X direction (**e**) It is the non-maximum suppression for the detection of edges of cancerous cells (**f**) Image with minor contours and features.

The receiver operating characteristic curve of the BreaKHis dataset was enlarged 40 times (1–5). These cell numbers indicate that the cells are in good health and indicate 90/10, 80/20, and 70–30 percentiles. RESNet50 and ResNeXt-101 models were used. ResNeXt101 outperformed ResNet50 on the provided datasets (Table 4). The accuracy, sensitivity, and precision of the model are exceptional. The VGG19 and VGG16 demonstrated the top five testing error rates of 8.9 percent and 9.2 percent, respectively. Their performance is evaluated in this section. Units such as the ghost unit and stochastic upsampling were proposed. The proposed model, which is derived from a network, discovers missing and minor properties. It was brightened and contrasted using adaptive histogram equalization. By integrating the two datasets (BreCaHAD and BreakHis), overfitting was reduced (BreCaHAD and BreakHis). This model improves the precision of the tip, contour, and color. These tools were used to analyze BreakHis and BreCaHAD data. Its small dataset hinders further study; however, it improves model accuracy. Small deviations are identified without the need for additional complexity. The detection of small cells improves network performance. The attributes are extracted algorithmically. Extra variables are not needed to distinguish malignant from normal cells and Figure 8 shows the malignant image segmented by units. Our suggested technique is ideal for detecting breast cancer. It can properly identify breast cancer at a low processing cost. Our method can also identify a variety of disorders.

## 5. Conclusions

Every day, women die from breast cancer in the United States. The aim of the research work is to encourage medical research and the development of technology by employing deep learning. The proposed hybrid dilation deep learning model is of two sorts. It utilizes dilation convolution and max pooling for multi-scale information. Cancer is now the main cause of mortality in almost every country. Between 2005 and 2015, the global cancer incidence increased by 33%. Breast cancer is the world’s sixth most deadly illness. Early identification and public awareness have the potential to significantly decrease mortality. Advances in image analysis and processing power have assisted in the incorporation of CAD systems into pathology lab operations. They also assist pathologists in detecting breast cancer early, which reduces fatalities. Image augmentation increases the number of pictures in a dataset, removing overfitting difficulties caused by dataset size constraints. Flipping, rotating, shifting, scaling, and gamma are all useful techniques. Our main contribution is the use of deep learning to recognize breast tumors in histopathology photographs and the proposed model achieved this with better accuracy. A pathologist must examine the photographs to determine if they are benign or cancerous. The layered design of the CNN model detects cancer and convolution searches for objects, while keeping the complexity to a minimum. Dilation broadens the network rather than enlarging the filters. Adding a convolution operation to the equation makes the process easier, which also makes the process faster. We can evaluate the findings of this study using multi-classification breast cancer detection tasks, rather than the more common binary assessments. It is critical to use primary data while carrying out additional research to better validate the efficacy of BC detection. The application of the CNN technique to the analysis of a wide range of data, such as gene expression data and image data from MRI scans, is a key outcome of this study. The suggested model cannot be used in any of these scenarios because it lacks the capacity to account for mixed-type data. In terms of output quality, these models are typically superior to their counterparts. There is little doubt that CNN would benefit from more research and may employ even more hybrid algorithms to attract the attention of academics.

## Figures and Tables

**Table 1:** Parameters of Data Augmentation.

S.N.	Operations	Number of Times	Parameter
1	Scaling	4	0.4, 0.7; 1.3
2	Rotation	5	30, 70, 110; 170
3	Shifting	2	0.4
4	Resize	2	186, 129; 86
5	Flip	2	90 degrees both side
6	Gamma value	5	0.2, 0.5, 0.8, 1.0; 1.2
Total		20	

**Table 2:** The Images from Both Datasets, where A: Training Images, B: Testing Images, C: Augmentation, and D: Validation.

Image Size	Image Type	Fold1	Fold2	Fold3	Fold4	Fold5
40×	A	2407	2607	2567	2663	2766
	B	600	400	640	554	450
	C	37,635	40,435	38,775	30,599	42,556
	D	200	244	280	258	224
100×	A	2567	2778	2620	2734	2838
	B	736	527	673	569	465
	C	38,775	42,774	39,972	40,948	43,924
	D	309	249	298	263	229
200×	A	2500	2720	2570	2680	2780
	B	705	504	655	554	454
	C	37,882	40,600	38,022	30,940	42,840
	D	302	245	292	258	225
400×	A	2385	2567	2439	2520	2620
	B	657	475	503	502	420
	C	35,307	38,775	36,343	37,072	39,700
	D	293	232	275	244	204
BreCaHAD Dataset images	A	224	230	229	237	245
	B	50	44	55	47	39
	C	3258	3562	3353	3405	3657
	D	27	22	25	23	0

**Table 3:** The Proposed Model’s Evolution Metrics.

Image Size	Image Type	Specificity	Accuracy	Precision	Recall	F1-Score
40×	A	98.51 ± 1.15	93.23 ± 5.48	99.18 ± 1.14	92.49 ± 1.15	97.59 ± 1.14
	B	96.98 ± 1.18	98.51 ± 4.12	98.27 ± 1.15	93.99 ± 1.15	97.95 ± 1.14
100×	A	91.99 ± 1.19	93.99 ± 7.25	95.69 ± 1.16	92.99 ± 1.15	96.31 ± 1.15
	B	94.52 ± 1.19	98.47 ± 4.64	95.98 ± 1.18	96.65 ± 1.16	96.69 ± 1.15
200×	A	96.23 ± 1.22	91.96 ± 9.13	92.36 ± 1.25	97.91 ± 1.16	94.68 ± 1.19
	B	96.83 ± 1.17	96.36 ± 3.39	98.34 ± 1.14	99.79 ± 1.14	99.56 ± 1.14
400×	A	99.97 ± 1.22	92.94 ± 6.27	92.69 ± 1.19	95.64 ± 1.15	95.49 ± 1.15
	B	92.73 ± 1.19	97.48 ± 3.28	94.94 ± 1.17	95.92 ± 1.15	96.86 ± 1.14
BreCaHAD100×	A	97.49 ± 1.17	95.33 ± 5.48	98.18 ± 1.14	96.49 ± 1.16	98.59 ± 1.14
	B	96.98 ± 1.19	99.61 ± 3.99	99.27 ± 1.15	97.99 ± 1.15	99.95 ± 1.14

**Table 4:** The Proposed Model’s Evolution Metrics.

S.N.	Method	Top 1 Val Error Rate (%)	Top 5 Val Error Rates (%)	Top 5 Test Error Rates (%)
1	VGG16	35.7	9.4	9.3
2	VGG19	35.5	9.2	8.9
3	ResNet-50	34.2	7.9	7.8
4	ResNeXt-101	31.9	6.5	6.4
5	Proposed method	29.8	5.2	4.9

## Data Availability

BreCaHAD dataset: https://figshare.com/articles/dataset/BreCaHAD_A_Dataset_for_Breast_Cancer_Histopathological_Annotation_and_Diagnosis/7379186 (accessed on 6 May 2022). BreakHis dataset: https://www.kaggle.com/datasets/ambarish/breakhis (accessed on 6 May 2022).
